# Sociodemographic profile of individuals eligible for lung cancer screening according to Brazilian guidelines: a national analysis

**DOI:** 10.36416/1806-3756/e20250391

**Published:** 2026-09-14

**Authors:** Ricardo Rodrigues Pereira, Samira Mariana de Campos Pereira

**Affiliations:** 1. Faculdade de Ciências Médicas de Minas Gerais - FCMMG - Belo Horizonte (MG) Brasil.; 2. Centro Universitário de Belo Horizonte, Belo Horizonte (MG) Brasil.

**Keywords:** Lung neoplasms, Mass screening, Tomography, X-ray computed, Socioeconomic factors, Cross-sectional studies

## Abstract

**Objective:**

To describe the sociodemographic profile of individuals eligible for lung cancer screening according to Brazilian guidelines.

**Methods:**

This was a cross-sectional study using data from the Brazilian Longitudinal Study of Aging. We included individuals 50-80 years of age, with a smoking history of ≥ 20 pack-years, who were either current smokers or former smokers who had quit within the past 15 years. Demographic, socioeconomic, and health characteristics were analyzed.

**Results:**

Among 8,946 participants, 577 were eligible. Most (57.4%) were male and 37.4% were between 55 and 59 years of age. Black and Brown (mixed-race) participants accounted for 53.5% of the sample, and 60.6% had < 9 years of schooling. Self-rated health was poor/fair in 52.5% of the individuals, 89.3% had no private health insurance, and 41.9% were in the lowest tertile for household income. Our findings show that eligible individuals often present features associated with social vulnerability, such as a low level of education, low income, and limited access to health care.

**Conclusions:**

These results highlight the importance of considering sociodemographic factors in the implementation of lung cancer screening strategies, to ensure equity in access and effectiveness of public policies aimed at early diagnosis.

## INTRODUCTION

Lung cancer is the leading cause of cancer-related mortality worldwide, accounting for approximately 1.8 million deaths annually, according to recent global estimates.^1^ In Brazil, it also ranks among the deadliest cancers, reflecting its aggressive nature and the high proportion of late-stage diagnoses.^2^ In this context, screening strategies that allow for early detection gain importance in public health debates. 

Large-scale multicenter studies, such as the National Lung Screening Trial (NLST) in the United States^3^ and the *Nederlands-Leuvens Longkanker Screenings Onderzoek* (NELSON, Dutch-Belgian Lung Cancer Screening Study) in Europe,^4^ demonstrated that screening with low-dose computed tomography (LDCT) reduces lung cancer mortality in high-risk populations, particularly among smokers and former smokers with heavy tobacco exposure. On the basis of these findings, several international guidelines were issued, such as those from the U.S. Preventive Services Task Force (USPSTF),^5^ which recommend annual LDCT screening for individuals 50-80 years of age, with a smoking history of ≥ 20 pack-years, who are current smokers or quit within the past 15 years. 

In 2023, the *Sociedade Brasileira de Pneumologia e Tisiologia* (SBPT, Brazilian Thoracic Society) and the *Colégio Brasileiro de Radiologia e Diagnóstico por Imagem* (CBR, Brazilian College of Radiology) published national guidelines with criteria similar to those of the USPSTF, adapted to the epidemiological context of Brazil.^6^ Those guidelines also define eligible individuals as those 50-80 years of age, with a smoking history of ≥ 20 pack-years, who are current smokers or quit within the past 15 years. 

Beyond the diagnostic accuracy of tests and the clinical eligibility criteria, effective implementation of screening programs requires understanding the sociodemographic and contextual characteristics of the target population. Factors such as education, income, access to health care, and self-perception of health directly affect adherence to examinations and follow-up, as well as the overall success of screening programs.^7^ ^,^ ^8^ The literature shows that vulnerable populations face systemic barriers that limit access to early diagnosis and timely treatment.^9^

In this scenario, analyzing the profile of the population considered eligible for lung cancer screening under the SBPT/CBR guidelines is essential to support equitable and effective public policies in Brazil. Studies such as the First and Second Brazilian Lung Cancer Screening Trials (BRELT1 and BRELT2, respectively) demonstrated the feasibility of LDCT screening in selected urban centers of the country, whereas analyses conducted more recently have highlighted operational barriers to eligibility identification and recruitment within routine care in the Brazilian *Sistema Único de Saúde* (SUS, Unified Health Care System).^10^ ^,^ ^11^ However, nationally representative data characterizing the sociodemographic and health profile of individuals eligible under the current Brazilian recommendations remain limited. Therefore, this study aimed to estimate the proportion of eligible individuals and examine lung cancer screening eligibility from a nationally representative sociodemographic perspective, characterizing the social, economic, and health profile of individuals considered eligible under the current SBPT/CBR guidelines.

## METHODS

This was a cross-sectional population-based study using data from the second wave of the *Estudo Longitudinal da Saúde dos Idosos Brasileiros* (ELSI-Brazil, Longitudinal Study of Health in Aging Brazilians), conducted between 2019 and 2021. The ELSI-Brazil was a national survey representative of the Brazilian population ≥ 50 years of age, based on multistage cluster sampling, stratified and calibrated by region, sex, age, and level of education. The dataset is publicly available and contains detailed information on sociodemographic, economic, behavioral, and health characteristics.^12^

### Sample

The individuals included were 50-80 years of age, were current smokers or former smokers who had quit within the past 15 years, and had a smoking history of ≥ 20 pack-years. Eligibility was determined in accordance with the 2023 SBPT/CBR guidelines for lung cancer screening.

### Variables

Smoking history was estimated from the self-reported average number of cigarettes smoked per day and years of smoking. For former smokers, the time since quitting was calculated to determine eligibility, with only those who had quit ≤ 15 years prior being considered eligible.

The following variables were described: sociodemographic characteristics, including sex, age (categorized into 5-year groups), self-reported race/skin color, level of education, region of Brazil, area of residence (urban/rural); economic characteristics, including household income (per capita tertile) and private health insurance (with/without); and self-rated health (SRH, categorized as good/very good/excellent or poor/fair). Eligibility was defined by combining age, smoking status, time since cessation, and smoking history (in pack-years). The variables were recoded and categorized for clarity and group comparisons.

### Data analysis

First, the proportion of eligible individuals 50-80 years of age in Brazil was estimated. The percentage distributions of key variables among eligible individuals were then calculated, with 95% confidence intervals. A significance level of α = 0.05 was adopted.

Analyses were conducted with the Stata statistical software package, version 17.0 (StataCorp LP, College Station, TX, USA), using survey commands, specifying primary sampling units (census tracts), stratification by municipality size, and individual sampling weights provided by the ELSI-Brazil, to account for the complexity of the sampling design.

### Ethical aspects

This study was exempt from submission to a research ethics committee, because it falls under Brazilian National Ministry of Health Resolution No. 510, of April 7, 2016, which regulates research involving publicly available secondary data with aggregated information that does not allow individual identification. The ELSI-Brazil study was approved by the Research Ethics Committee of Fundação Oswaldo Cruz (Reference no. 34649814.3.0000.5091), in the city of Belo Horizonte, Brazil.

## RESULTS

Of the 8,946 participants 50-80 years of age in the ELSI-Brazil cohort (Table 1), 577 were considered eligible for lung cancer screening based on the SBPT/CBR criteria. This corresponds to 7.1% (95% CI: 6.1-8.2) of the weighted national sample.

**Table 1:** Sociodemographic and health profile of individuals eligible for lung cancer screening according to the Brazilian guidelines.^a^

Variable	%	95% CI
Sex		
Female	42.6	37.6-47.9
Male	57.4	52.1-62.4
Age (years)		
50-54	20.6	15.5-26.8
55-59	37.4	31.2-43.9
60-64	17.3	13.7-21.7
65-69	12.4	9.7-15.7
70-74	6.7	4.8-9.3
75-80	5.5	3.9-7.8
Race/skin color		
White	46.0	38.5-53.8
Black/Brown (mixed)	53.5	45.8-61.1
Asian/Indigenous	0.5	0.1-1.5
Schooling		
< 9 years	60.6	54.1-66.7
9 years	9.2	6.7-12.3
> 9 years < 12	4.6	2.7-7.6
12 years	22.0	17.0-28.0
> 12 years	3.7	2.2-6.2
Region		
North	4.02	1.84-8.57
Northeast	31.05	19.95-44.86
Southeast	43.42	30.68-57.08
South	13.32	6.59-25.08
Central-west	8.19	3.87-16.52
Area of residence		
Rural	15.65	10.58-22.53
Urban	84.35	77.47-89.42
Self-rated health		
Poor/fair	52.5	45.8-59.1
Good/very good/excellent	47.5	40.9-54.2
Household income (tertile)		
Low	41.9	34.5-49.7
Middle	29.9	24.5-35.9
High	28.2	22.7-34.5
Private health insurance		
No	89.3	85.6-92.2
Yes	10.7	7.8-14.4

a Brazilian Thoracic Society/Brazilian College of Radiology guidelines (Brazilian Longitudinal Study of Aging, 2019-2021).

Of the eligible individuals, 57.4% (95% CI: 52.1-62.4) were male. The mean age was 60.3 ± 7.7 years. The dominant age group was 55-59 years (37.4%; 95% CI: 31.2-43.9), followed by 50-54 years (20.6%; 95% CI: 15.5-26.8), 60-64 years (17.3%; 95% CI: 13.7-21.7), 65-69 years (12.4%; 95% CI: 9.7-15.7), 70-74 years (6.7%; 95% CI: 4.8-9.3), and 75-80 years (5.5%; 95% CI: 3.9-7.8). 

Regarding race/skin color, 53.5% (95% CI: 45.8-61.1) of the eligible individuals identified as Black or Brown (mixed), 46.0% (95% CI: 38.5-53.8) identified as White, and only 0.5% (95% CI: 0.1-1.5) identified as Asian or Indigenous.

Regarding education, most of the eligible individuals had a low level of schooling: 60.6% (95% CI: 54.1-66.7) had fewer than 9 years of schooling, 9.2% (95% CI: 6.7-12.3) had 9 years , 4.6% (95% CI: 2.7-7.6) had 10-11 years, 22.0% (95% CI: 17.0-28.0) had 12 years, and 3.7% (95% CI: 2.2-6.2) had > 12 years of schooling.

The geographic distribution of eligible individuals varied across regions of Brazil. The highest proportion was observed in the southeast (43.4%; 95% CI: 30.7-57.1), followed by the northeast (31.1%; 95% CI: 20.0-44.9), south (13.3%; 95% CI: 6.6-25.1), central-west (8.2%; 95% CI: 3.9-16.5), and north (4.0%; 95% CI: 1.8-8.6). With regard to area of residency, most eligible individuals (84.4%; 95% CI: 77.5-89.4) lived in urban areas.

Most eligible individuals (89.3%; 95% CI: 85.6-92.2) lacked private health insurance, with only 10.7% (95% CI: 7.8-14.4) having coverage. Regarding SRH, 52.5% (95% CI: 45.8-59.1) the eligible individuals reported poor or fair health,. Of the eligible individuals, 41.9% (95% CI: 34.5-49.7) were in the lowest tertile of household income, 29.9% (95% CI: 24.5-35.9) were in the middle tertile, and 28.2% (95% CI: 22.7-34.5) were in the highest tertile.

## DISCUSSION

The sociodemographic profile of individuals eligible for lung cancer screening according to SBPT/CBR criteria revealed key markers of social vulnerability. The fact that only 7.1% of the sample met the eligibility criteria should not be seen as a limitation of screening, but rather as a reflection of strict guidelines designed to balance benefit, cost-effectiveness, and safety, as demonstrated in international studies like the NLST and NELSON.^3^ ^,^ ^4^

The high proportion of eligible individuals without private health insurance (89.3%) underscores the heavy reliance on the SUS. That dependence poses significant challenges, given the unequal distribution of diagnostic imaging services in Brazil, particularly LDCT, the availability of which remains limited in many regions. This aligns with SUS diagnostic coverage data showing inequalities in CT access in suburban, rural, and low-income areas.^13^ ^-^ ^15^

The predominance of individuals with a low level of education (60.6%) and of Black/Brown (mixed) individuals, who accounted for 60.6% and 53.5% of the sample, respectively, highlights the role of educational and racial inequalities as social determinants of health. Prior studies have shown that people with less schooling have less knowledge about cancer symptoms, lower access to preventive examinations, and longer delays before diagnosis.^16^ ^,^ ^17^ Race/skin color has also been linked to worse lung cancer outcomes in the United States and Latin America.^18^

The concentration of eligible individuals in the 55- to 64-year age group is consistent with the period of greatest accumulated smoking exposure before cessation. More than half (52.5%) of the eligible individuals rated their health as poor/fair, suggesting a higher prevalence of chronic comorbidities. In other populations, that perception has been associated with lower adherence to screening, representing an additional barrier to care.^16^ In epidemiological research, SRH is commonly categorized as “poor/fair” or “good/very good/excellent”, a classification that has been consistently validated as a robust global health indicator. Studies have demonstrated that chronic conditions, functional limitations, and increased health care utilization are more common among individuals reporting poor or fair health. In addition, SRH has been shown to be an independent predictor of mortality across different populations. A meta-analysis including multiple cohort studies demonstrated that the mortality risk is significantly higher among individuals reporting poor health than among those reporting excellent health.^19^ Similarly, longitudinal analyses from the Brazilian Pró-Saúde cohort^20^ and large population-based studies conducted in the United Kingdom^21^ confirmed that poor/fair SRH is strongly associated with adverse clinical outcomes, even after adjustment for objective health measures and sociodemographic factors. In this context, the high proportion of poor/fair SRH observed among screening-eligible individuals may reflect a substantial multimorbidity burden and increased vulnerability within this population. Such clinical and perceived health vulnerability, particularly when combined with low educational attainment and the logistical demands of repeated screening visits, may represent additional barriers to adherence and follow-up, as previously observed in lung cancer screening settings.^22^

These findings align with those of studies such as the BRELT1 and BRELT2. In the BRELT1, conducted in a region endemic for granulomatous diseases, detection rates were comparable to those in the international literature despite diagnostic challenges from benign findings on LDCT.^10^ Similarly, the sociodemographic profile in the BRELT2 showed a predominance of low-income, low-education individuals, highlighting the need for tailored approaches to ensure successful implementation of lung cancer screening in Brazil.^11^

Beyond individual-level characteristics, the geographic and territorial distribution of the eligible population is a critical dimension for the implementation of lung cancer screening in Brazil. In our analysis, a substantial proportion of eligible individuals resided in the southeast (43.4%) and northeast (31.0%), whereas the north and central-west accounted for smaller proportions. These findings gain further relevance when considered alongside the marked regional disparities in the availability of computed tomography (CT) scanners across the country. National data indicate that CT equipment is disproportionately concentrated in the southeast and south, with substantially lower availability per capita in the north and central-west, particularly within the SUS. In addition, only a minority of Brazilian municipalities have CT scanners, reflecting a strong concentration of imaging services in larger urban centers.^23^ ^,^ ^24^ Such structural inequalities may limit geographic access to LDCT, especially for rural and socially vulnerable populations, potentially exacerbating delays in screening initiation and follow-up.^23^ Therefore, the regional distribution of individuals eligible for lung cancer screening must be interpreted in light of the unequal regional capacity to deliver diagnostic imaging services, underscoring the need for implementation strategies that address epidemiological demand and structural supply constraints within the SUS.

One limitation of this study is that smoking status, cumulative exposure (pack-years), and time since cessation were entirely self-reported. Self-reported tobacco use may be subject to recall bias, particularly when estimating lifetime pack-year exposure, and to social desirability bias, potentially leading to underreporting of smoking intensity or duration. Misclassification of cumulative exposure could affect the identification of individuals meeting screening eligibility criteria. However, self-reported smoking status has demonstrated acceptable validity in epidemiological studies when compared with biochemical verification methods, including cotinine assays.^25^ ^-^ ^27^ Systematic reviews report generally high sensitivity and specificity of self-reported smoking status in population-based surveys.^27^ In this context, any underestimation of smoking exposure would likely result in conservative estimates of lung cancer screening eligibility rather than overestimation. Nevertheless, this limitation should be considered when interpreting the absolute prevalence of eligible individuals.

The sociodemographic profile of individuals eligible for lung cancer screening in Brazil, under the SBPT/CBR criteria, reveals a group largely composed of people with a low level of education, predominantly Black or Brown (of mixed race), without private health insurance, and with negative SRH. These findings reflect long-standing social inequities that have historically shaped health care access in Brazil. Beyond defining who meets the clinical eligibility criteria, this profile has important implications for screening adherence and continuity of care. Socioeconomic vulnerability could influence the entire screening pathway, from initial uptake to follow-up of abnormal findings. Individuals with lower educational attainment and reduced access to resources could face barriers related to health literacy, transportation, competing work demands, and fragmented care. Recent studies have documented low adherence to annual lung cancer screening among socially disadvantaged populations.^28^ In addition, socioeconomic and demographic determinants have been identified as significant predictors of continued participation and follow-up completion.^29^ Furthermore, racial disparities in adherence to annual screening and recommended follow-up care have been observed in multicenter cohort studies.^30^ These inequities can attenuate the potential mortality benefits of screening if not explicitly addressed in implementation strategies. 

Therefore, identifying a socially vulnerable eligible population underscores the need for approaches within the SUS that go beyond clinical risk stratification, incorporating patient navigation, strengthened coordination with primary care, and targeted communication strategies to promote equitable adherence and follow-up. Importantly, clinical eligibility and programmatic feasibility represent distinct dimensions of screening policy. Although eligibility criteria define who should be screened on the basis of epidemiological risk, feasibility depends on the health system’s capacity to operationalize these recommendations through adequate infrastructure, provider engagement, referral pathways, and sustained follow-up mechanisms. Recent implementation studies in lung cancer screening have demonstrated that organizational barriers, limited provider awareness, workflow constraints, and fragmented care coordination can substantially reduce screening uptake and continuity of care even when eligibility criteria are well established.^31^ ^,^ ^32^ These findings support the assertion that epidemiological demand must be interpreted alongside system-level readiness. In Brazil, where access to imaging and specialist care varies across regions, expanding lung cancer screening will require aligning eligibility criteria with the actual capacity of the health system to deliver timely diagnoses and follow-up within the SUS.

## References

[1] Sung H, Ferlay J, Siegel RL, Laversanne M, Soerjomataram I, Jemal A (2020). Global Cancer. Statistics.

[2] Instituto Nacional de Câncer (2022). Estimativa 2023: Incidência de câncer no Brasil.

[3] Aberle DR, Adams AM, Berg CD, Black WC, Clapp JD, Fagerstrom RM (2011). Reduced lung-cancer mortality with low-dose computed tomographic screening. N Engl J Med.

[4] de Koning HJ, van der Aalst CM, de Jong PA, Scholten ET, Nackaerts K, Heuvelmans MA (2020). Reduced lung-cancer mortality with volume CT screening in a randomized trial. N Engl J Med.

[5] U.S. Preventive Services Task Force (2021). Screening for lung cancer: recommendation statement. JAMA.

[6] Sociedade Brasileira de Pneumologia e TisiologiaColégio Brasileiro de Radiologia (2023). Diretriz Brasileira para Rastreamento do Câncer de Pulmão.

[7] Sosa E, D'Souza G, Akhtar A, Sur M, Love K, Duffels J (2021). Racial and socioeconomic disparities in lung cancer screening in the United States A systematic review. CA Cancer J Clin.

[8] Sousa EL, Rodrigues FDCA, Saraiva JGC, Santos RSM, Zagury DGM, Brito GE (2025). Geographic disparities and temporal trends regarding access to cancer treatment a spatial analysis, Brazil, 2015-2022. Epidemiol Serv Saude.

[9] Mehta AJ, Stock S, Gray SW, Nerenz DR, Ayanian JZ, Keating NL (2018). Factors contributing to disparities in mortality among patients with non-small-cell lung cancer. Cancer Med.

[10] dos Santos RS, Franceschini JP, Chate RC, Ghefter MC, Kay F, Trajano AL (2016). Do Current Lung Cancer Screening Guidelines Apply for Populations With High Prevalence of Granulomatous Disease Results From the First Brazilian Lung Cancer Screening Trial (BRELT1). Ann Thorac Surg.

[11] Hochhegger B, Marchiori E, Irion K (2022). Challenges of implementing lung cancer screening in a developing country results of BRELT2. JCO Glob Oncol.

[12] Lima-Costa MF, de Andrade FB, de Souza PRB, Neri AL, Duarte YAO, Castro-Costa E (2018). The Brazilian Longitudinal Study of Aging (ELSI-Brazil) objectives and design. Am J Epidemiol.

[13] Svartman FM, Azambuja MI, Palma EA, Sartori APG, Leite MMR (2024). Lung cancer screening eligibility and recruitment during routine care by pulmonologists barriers and new opportunities in the Brazilian public healthcare system. J Bras Pneumol.

[14] Silva GAE, Souza-Júnior PRB, Damacena GN, Szwarcwald CL (2017). Early detection of breast cancer in Brazil data from the National Health Survey, 2013. Rev Saude Publica.

[15] Alencar CAC, de Oliveira DC, Teixeira ABM, Lemos LMG, Quesado RCS, Santos IMA (2024). Computed tomography and magnetic resonance imaging in Brazil an epidemiological study on the distribution of equipment and frequency of examinations, with comparisons between the public and private sectors. Radiol Bras.

[16] antos EFS, Monteiro CN, Vale DB, Louvison M, Goldbaum M, Cesar CLG (2023). Social inequalities in access to cancer screening and early detection A population-based study in the city of São Paulo, Brazil. Clinics (Sao Paulo).

[17] Lofters AK, Telner D, Kalia S, Slater M (2018). Association Between Adherence to Cancer Screening and Knowledge of Screening Guidelines. JMIR Cancer.

[18] Ryan BM (2018). Lung cancer health disparities. Carcinogenesis.

[19] DeSalvo KB, Bloser N, Reynolds K, He J, Muntner P (2006). Mortality prediction with a single general self-rated health question a meta-analysis. J Gen Intern Med.

[20] Nery Guimarães JM, Chor D, Werneck GL, Carvalho MS, Coeli CM, Lopes CS (2012). Association between self-rated health and mortality 10 years follow-up to the Pró-Saúde cohort study. BMC Public Health.

[21] Mutz J, Lewis CM (2022). Cross-classification between self-rated health and health status longitudinal analyses of all-cause mortality and leading causes of death in the UK. Sci Rep.

[22] Quaife SL, Janes SM, Brain KE (2021). The person behind the nodule a narrative review of the psychological impact of lung cancer screening. Transl Lung Cancer Res.

[23] Alencar CAC, de Oliveira DC, Teixeira ABM, Lemos LMG, Quesado RCS, Santos IMA (2024). Computed tomography and magnetic resonance imaging in Brazil an epidemiological study on the distribution of equipment and frequency of examinations, with comparisons between the public and private sectors. Radiol Bras.

[24] Santos DL, Leite HJ, Rasella D, Silva SA (2014). CT scanners in the Brazilian Unified National Health System installed capacity and utilization [Article in Portuguese]. Cad Saude. Publica.

[25] Patrick DL, Cheadle A, Thompson DC, Diehr P, Koepsell T, Kinne S (1994). The validity of self-reported smoking a review and meta-analysis. Am J Public Health.

[26] Vartiainen E, Seppälä T, Lillsunde P, Puska P (2002). Validation of self reported smoking by serum cotinine measurement in a community-based study. J Epidemiol Community Health.

[27] Connor Gorber S, Schofield-Hurwitz S, Hardt J, Levasseur G, Tremblay M (2009). The accuracy of self-reported smoking a systematic review of the relationship between self-reported and cotinine-assessed smoking status. Nicotine Tob Res.

[28] Parikh MJ, Chai LF, Russo MG, Tompkins AK, Akinade O, Erkmen CP (2025). Dismal adherence to lung cancer screening in a diverse urban population. J Thorac Cardiovasc Surg.

[29] Wang Z, Kim Y, Mortani Barbosa EJ (2024). Demographics and socioeconomic determinants of health predict continued participation in a CT lung cancer screening program. Curr Probl Diagn Radiol.

[30] Kim RY, Rendle KA, Mitra N, Saia CA, Neslund-Dudas C, Greenlee RT (2022). Racial disparities in adherence to annual lung cancer screening. Ann Am Thorac Soc.

[31] Golden SE, Currier JJ, Ramalingam N (2024). Primary Care Providers Experiences Implementing Low-Dose Computed Tomography Recommendations for Lung Cancer Screening. J Am Board Fam Med.

[32] Spalluto LB, Lewis JA, Stolldorf D, Yeh VM, Callaway-Lane C, Wiener RS (2021). Organizational Readiness for Lung Cancer Screening A Cross-Sectional Evaluation at a Veterans Affairs Medical Center. J Am Coll Radiol.

